# Similarity in Transcytosis of nNOSα in Enteric Nerve Terminals and Beta Cells of Pancreatic Islet

**DOI:** 10.3389/fmed.2014.00020

**Published:** 2014-07-31

**Authors:** Arun Chaudhury

**Affiliations:** ^1^Division of Surgery, Brigham and Women’s Hospital, Harvard Medical School and VA Boston HealthCare System, Boston, MA, USA

**Keywords:** nNOS, myosin Va, diabetes, dimer, LC8

Transcytosis of proteins and hydrodynamic flow of cytoplasm is a major mechanism to sustain physiology in all cells, observable from gametes (1) to mature adult cells and tissues (2, 3). Mammalian cells involved in secretion discretely need to respond to the environment and move components within the cells and position them at appropriate locations for secretion (4, 5). This process involves force generation using Gibbs free energy of hydrolysis of adenosine triphosphate (ATP). The ATPase is most often myosin, a naturally occurring cellular ATPase known for its wide role in generation of cellular force (6). The nanomechanics of transport involve the necessary target cargoes, in association with myosin and track on actin filaments, which are ubiquitous cellular cytoskeletal scaffolds of metazoan cells (7). Cellular secretion encompasses multiple physiological systems operating on wide range of time scales including the processes of exocrine and endocrine glandular secretions and neuronal secretion in response to discrete electrical field stimulation, commonly referred to as neurotransmission (8, 9).

Here, similarity is outlined between the mechanisms involved in gaseous nitric oxide (NO) synthesis within the enteric nerve terminals in response to an action potential (10–15) and during glucose sensing and insulin granule exocytosis by pancreatic beta cells (16–20) (Figure 1). These comparisons provide new directions to investigate physiology of insulin exocytosis in health and potential dysfunction as a pathophysiologic mechanism of diabetes mellitus. NO may perform physiological functions during insulin exocytosis from large dense core secretory vesicles (LDCVs). Reasonable evidence and consensus exist regarding the role of NO during biphasic secretion of insulin under normal physiological conditions (16, 17, 21–24). Though the exact contribution of NO is not well defined, incipient convincing evidence exists regarding *de novo* synthesized NO by neuronal nitric oxide synthase (nNOS) to maintain a pool of glucokinase in association with insulin secretory granules (25). Glucokinase, a form of low-sensitive hexokinase, catalyzes the first and rate-limiting step in conversion of glucose to a hexose phosphate, which sets a feedback balance between sensing the extracellular glucose concentration and operating this as a stimulus for insulin granules’ exocytosis. It was reported from early studies that infusion of l-arginine increases insulin release (26, 27), and this is disrupted in patients with non-insulin-dependent diabetes mellitus (NIDDM) (28). Incipient evidence also exists regarding the role of l-citrulline in replenishing cellular levels of l-arginine through arginosuccinate and restoring beta cell function (29).

**Figure 1:**
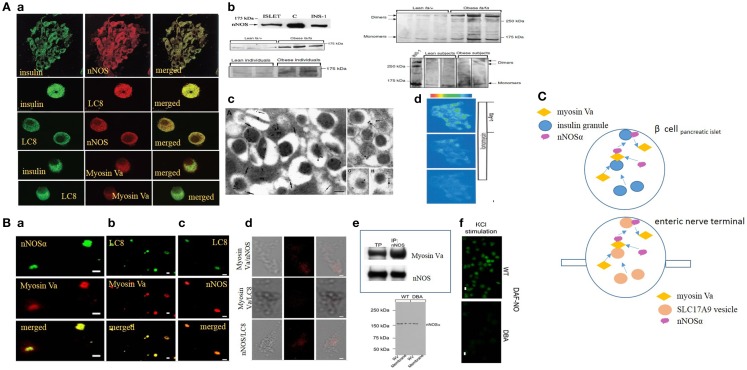
**(A)** (a) Imaging studies of colocalization of nNOS, LC8, and myosin Va with insulin granules in pancreatic islets. Upper panel, pancreatic islets stained for insulin colabel with neuronal nitric oxide synthase (nNOS). Isolated islets colabel for insulin and LC8 (middle panel), nNOS and LC8 (third panel), insulin and myosin Va (fourth panel), and LC8 and myosin Va (bottom panel). (b) nNOS immunoblots in normal and diseased islets. Islets and INS1 cell line label for ~160 kDa nNOS band. Lower panels on the left show increased nNOS bands on the western blots obtained from fa/fa Zucker obese rats and obese human individuals, models of insulin hypersecretor phenotypes. The right panels show that nNOS exists as a dimer, revealed by cold SDS-PAGE. Dimer/monomer ratios are raised in the hypersecretor phenotypes. (c) Electron micrographs of insulin LDCVs (secretory granules). (a,e) Electron micrographs showing immune particles representing insulin and nNOS. In (e), note nNOS on the membrane of the LDCV. (g,h) Of the electron micrographs show nNOS-LC8 in the core and membrane of insulin LDCVs. (d) Ionomycin and l-arginine enhances NO production in INS1 cell lines, imaged by loaded diaminofluorescein. [Figures modified with permission from Lajoix et al. (17), Mezghenna et al. (16) and Smukler et al. (22).] **(B)** (a–c) Imaging studies of colocalization of nNOS, LC8, and myosin Va in isolated enteric synaptosomes. (d) Proximity ligation assay (PLA) shows blobs of protein interactions of nNOS, LC8, and myosin Va in isolated enteric synaptosomes. (e) Upper panel shows co-immunoprecipitation of nNOS-myosin Va in mice stomach lysate; lower panel shows intact nNOSα in whole varicosities of wild type and DBA/2J dilute mice, but absence of membrane bound nNOSα in DBA/2J, indicating the potential role of myosin Va in membrane transposition of nNOS. (f) KCl stimulation of plated varicosities shows significantly reduced DAF-NO signal in enteric synaptosomes obtained from DBA/2J mice, in comparison to C57BL/6J mice. [Figures modified with permission from Chaudhury et al. (11, 12).] **(C)** Cartoon depicting similarity in mechanisms of transcytosis of insulin and nNOS by myosin Va in beta cells and enteric synaptosomes. Note the similarity of organization of non-vesicular nNOS with either SLC17A9 purinergic vesicles within nerve terminals or insulin granules in beta cells of islets of pancreas. Genomic inhibition of myosin Va may be a potential initial upstream pathophysiologic mechanism contributing to both progression of diabetes by impairing insulin exocytosis, as well as causing multiorgan dysfunction, for example, reduction of inhibitory nitrergic neuromuscular transmission in the gut. Arrows are shown to indicate directionality of movements.

In the smooth muscle-*en passant* nerve terminal junctions in the gastrointestinal tract, inhibitory neurotransmission involves release of vesicular ATP and instantaneously synthesized gaseous NO (30, 31). This kind of tandem transmission involving precision release of a vesicular and a non-vesicular neurotransmitter is the one of its kind only example in the body. Importantly, the contribution of nitrergic component is critical to inhibitory neurotransmission, as loss of nitrergic synthesis results in failure of mechanical relaxations and manifestations of stasis of luminal contents like gastroparesis. NO synthesis is facilitated by the alpha isoform of nNOS, which has the potential to bind to membrane by cysteine dimerization of its N-terminal domain with palmitoyl-PSD95 (13). Examples from numerous systems suggest the general feature that membrane localization of nNOSα is perhaps critical for its function. Though cytosolic nNOSα may exist as a dimer and technically can favor electron transfer during oxidation of l-arginine for NO synthesis, it seems that proximity to calcium sources such as the calcium channel may be an important requirement for membrane transposition for optimal nNOSα enzymatic activity (13–15). Furthermore, it has been demonstrated that cytosolic nNOS is phosphorylated at serine847, which prevents calmodulin interaction and positive allostery during neurotransmission (13, 14). Recent evidence has shown the role of unconventional motor proteins like myosin Va in membrane transport of nNOSα within nerve terminals (12). Hypomorphic mutant DBA/2J mice lacking functional myosin Va shows evidence of impaired prejunctional NO synthesis and NO-mediated smooth muscle responses including slow IJP and mechanical relaxations (11, 12).

Reliable evidence exists that in the beta cells of the pancreas, nNOS alpha isoform exists (17, 18). This is seen in rat, mice, and human islets (32). nNOS alpha dimer binds to the core of insulin granules and also concentrated in the subterminal membranes (17). The beta cells also contain the light chain of dynein, LC8, earlier referred to as protein inhibitor of nNOS (PIN) (17). In enteric neuronal varicosities, LC8 facilitates nNOS-myosin Va protein interactions, confirmed independently by the traditional co-immunoprecipitation experiments and visually by proximity ligation assay (PLA) (11, 12). Myosin Va has been demonstrated in the pancreatic beta cells, colocalized with insulin and PIN (LC8) (17). Given these comparative levels of evidence, it may be reasonable to speculate that transcellular movements of nNOS within beta cells of pancreatic islets and translocation to the subcortical zone likely involves myosin Va, though this has never been directly demonstrated.

There are seven exonic regions (A–G) in the N-terminal portion of the tail region of myosin Va that facilitates cargo binding (33). For example, in the skin, the melanocytes have ACDE and lacks B exon. In neuronal cells, the exonic region is represented as ABE. Notably, the B region comprising only three bases, representing amino acids 1282–1284 of myosin Va, which represents the region for interaction of myosin Va with nNOS via LC8. In pancreatic beta cells, the exonic component is similar to brain myosin Va (34), providing the likelihood that nNOS-LC8 binds with myosin Va, though any direct evidence for this is lacking.

In DBA/2J mice, prejunctional nitrergic synthesis during enteric nerve-smooth muscle neurotransmission has been demonstrated to be significantly reduced (11, 12). It seems likely that NO synthesis in pancreatic beta cells may be diminished in DBA/2J mice, though this remains to be tested. Whether inhibition or reduction of NO synthesis results in impaired glucose tolerance or frank diabetes is not known for DBA mice, though streptozotocin injection in DBA/2J has been used as a model of peripheral neuropathy (35). The role of myosin Va in secretory granule exocytosis (36), including insulin granules (37, 38), has been reported. It may be hypothesized that DBA phenotype should predispose to a diabetic state. As anticipated, it has been reported that DBA loci confers increased risk of diabetes (39). In the initial phases, there is a hypersecretor phenotype of C57BLKS/J mice created on a DBA background, with increased secretion of insulin. This has been reported to result from defects in nicotinamide nucleotide transhydrogenase (Nnt), resulting in diminution of reducing potentials and increased oxidative stress (40, 41), as well as other defects like that of amino acid l-arginine transporter SLC7A3, which may result in defective NO synthesis. The hypersecretor phenotype seen in the early stages of DBA/2J mice (42) may represent a prediabetic condition. This may ultimately contribute to exhaustion of insulin in the islets and frank manifestation of insulin-dependent diabetes in DBA/2J mice. Myosin Va facilitates transcellular movement of glucose transporters like GLUT4, which are important components for mobilization of glucose in the peripheral organs like the skeletal muscles and adipocytes (43–46). It is possible that the initial phases of DBA/2J might represent a prediabetic state and a condition of peripheral insulin resistance resulting from impaired or suboptimal mobilization of myosin Va-dependent glucose transporters like GLUT4 results in the hypersecretor phenotype of the pancreatic islets. Temporal studies using DBA mice shall provide insights into the progression of prediabetic state to one of frank diabetes mellitus and complications arising as a result of long-standing diabetes.

Myosin Va has been shown to facilitate both the first phase of insulin release, as well as during sustained phase when storage pool vesicles are recruited to a readily releasable pool in a non-linear dynamics (45, 46). This may occur due to facilitator effect on insulin-containing LDCV movement in the cell cortex. Though it has not been specifically tested, it is likely that myosin Va facilitates both secretory granule vesicular movement, as well as nNOS movement toward the cell periphery for association with insulin granules. In obese Zucker rats and islets derived from obese humans, it has been shown that these islets demonstrate a hypersecretor phenotype, and has been related to increased nNOS dimers (16). Recent observation has been made regarding significant reduction of myosin Va in myenteric neuronal soma and nerve varicosities of jejunum in streptozotocin-induced diabetes, likely a result of inhibition of genomic transcription of myosin Va (47). The reduction in myosin Va may result from reduction in its glucose-sensitive transcription factor Snail (48). It may be worthwhile to examine whether hyperglycemia globally affects this transcription factor, which in turn may affect all myosin Va-related functions including nNOS enzymatic activity during enteric nitrergic neurotransmission and insulin granule exocytosis and its regulation in beta cells of pancreas.

## Conflict of Interest Statement

The author declares that the research was conducted in the absence of any commercial or financial relationships that could be construed as a potential conflict of interest.
